# Diaqua­bis(ciprofloxacinato)manganese(II) 2,2′-bipyridine solvate tetrahydrate

**DOI:** 10.1107/S1600536809021783

**Published:** 2009-06-17

**Authors:** Yan-Jun Wang, Na Wang, Rui-Ding Hu, Qiu-Yue Lin, Yun-Yun Wang

**Affiliations:** a Zhejiang Key Laboratory for Reactive Chemistry on Solid Surfaces, Institute of Physical Chemistry, Zhejiang Normal University, Jinhua, Zhejiang 321004, People’s Republic of China, and, College of Chemistry and Life Science, Zhejiang Normal University, Jinhua 321004, Zhejiang, People’s Republic of China

## Abstract

In the crystal structure of the title compound {systematic name: diaquabis­[1-cyclo­propyl-6-fluoro-4-oxo-7-(piperazin-1-yl)-1,4-dihydro­quinoline-3-carboxyl­ato]manganese(II) 2,2′-bi­pyridine solvate tetrahydrate}, [Mn(C_17_H_17_FN_3_O_3_)_2_(H_2_O)_2_]·C_10_H_8_N_2_·4H_2_O, the pyridone O and one carboxyl­ate O atom of the two ciprofloxacin ligands are bound to the Mn^II^ ion and occupy the equatorial positions, while the two aqua O atoms lie in the apical positions resulting in a distorted octa­hedral geometry. The crystal packing is stabilized by N–H⋯O and O–H⋯O hydrogen bonding interactions.

## Related literature

Manganese is a cofactor or required metal ion for many enzymes, such as superoxide dismutase, glutamine synthetase and arginase, see: Dukhande *et al.* (2006 ▶). 
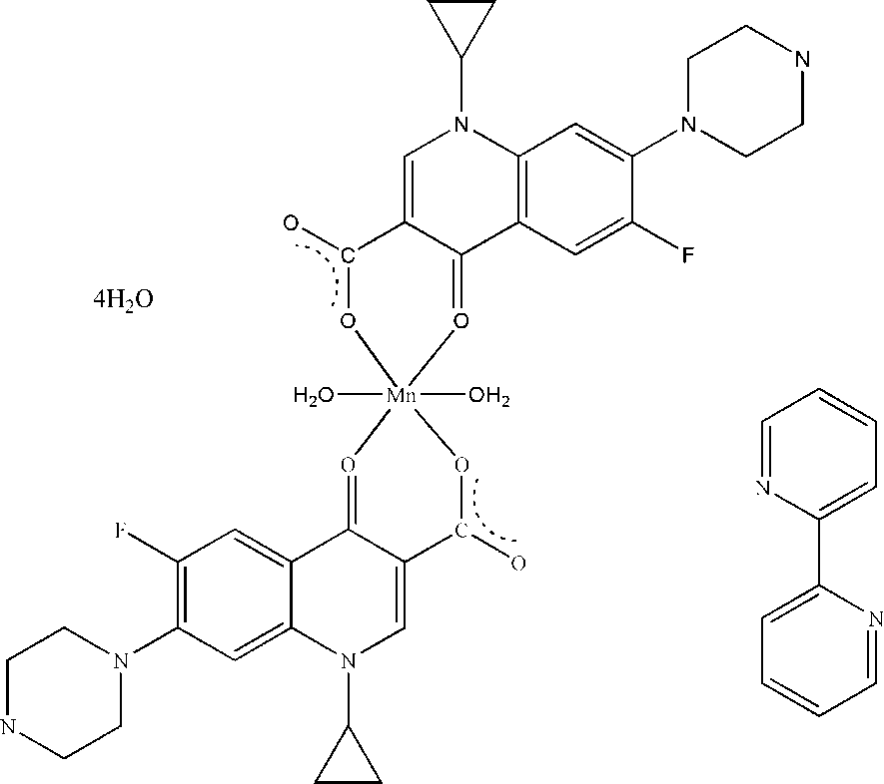


## Experimental

### 

#### Crystal data

[Mn(C_17_H_17_FN_3_O_3_)_2_(H_2_O)_2_]·C_10_H_8_N_2_·4H_2_O
*M* *_r_* = 979.89
Triclinic, 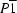

*a* = 10.0355 (3) Å
*b* = 11.1409 (3) Å
*c* = 11.8461 (3) Å
α = 66.905 (2)°
β = 68.933 (2)°
γ = 85.858 (2)°
*V* = 1133.22 (6) Å^3^
*Z* = 1
Mo *K*α radiation
μ = 0.37 mm^−1^
*T* = 296 K
0.42 × 0.17 × 0.05 mm

#### Data collection

Bruker APEXII CCD area-detector diffractometer
Absorption correction: multi-scan (*SADABS*; Sheldrick, 1996 ▶) *T*_min_ = 0.926, *T*_max_ = 0.983
14989 measured reflections
5061 independent reflections
3310 reflections with *I* > 2σ(*I*)
*R*_int_ = 0.042

#### Refinement

*R*[*F* ^2^ > 2σ(*F* ^2^)] = 0.084
*wR*(*F* ^2^) = 0.274
*S* = 1.08
5061 reflections
313 parameters
6 restraints
H atoms treated by a mixture of independent and constrained refinement
Δρ_max_ = 1.07 e Å^−3^
Δρ_min_ = −0.65 e Å^−3^

### 

Data collection: *APEX2* (Bruker, 2004 ▶); cell refinement: *SAINT* (Bruker, 2004 ▶); data reduction: *SAINT*; program(s) used to solve structure: *SHELXTL* (Sheldrick, 2008 ▶); program(s) used to refine structure: *SHELXTL*; molecular graphics: *SHELXTL*; software used to prepare material for publication: *SHELXTL*.

## Supplementary Material

Crystal structure: contains datablocks I, global. DOI: 10.1107/S1600536809021783/at2799sup1.cif

Structure factors: contains datablocks I. DOI: 10.1107/S1600536809021783/at2799Isup2.hkl

Additional supplementary materials:  crystallographic information; 3D view; checkCIF report

## Figures and Tables

**Table 1:** Hydrogen-bond geometry (Å, °)

*D*—H⋯*A*	*D*—H	H⋯*A*	*D*⋯*A*	*D*—H⋯*A*
N3—H3*B*⋯O3^i^	0.86	2.22	2.661 (5)	112
O1*W*—H1*WA*⋯N4^ii^	0.86 (2)	2.03 (2)	2.880 (6)	171 (7)
O3*W*—H3*WB*⋯O2*W*^ii^	0.85	2.13	2.910 (4)	153
O1*W*—H1*WB*⋯O3*W*	0.86 (2)	2.15 (2)	3.009 (5)	170 (6)
O2*W*—H2*WA*⋯N3	0.765 (18)	2.53 (3)	3.125 (5)	136 (4)

Symmetry codes: (i) 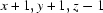; (ii) 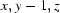.

**Fig. 1.**
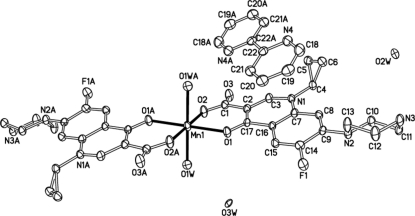
A view of the molecule of (I) showing the atom-labelling scheme with displacement ellipsoids drawn at the 30% probability.

## supplementary crystallographic information

### Comment

1-Cyclopropyl-6-fluoro-1,4-dihydro-4-oxo-7-(1-piperazinyl)-3-quinoline carboxylic acid hydrochloride (ciprofloxacin hydrochloride), is the third generation quinolone antibacterial drug with broad-spectrum antibacterial activity, especially aerobic gram-negative bacilli high antibacterial activity. It can interfere the synthesis of DNA, destroy the fission of cells in order to sterilize by inhibiting DNA gyrase. Manganese is an important trace element needed for normal physiological functions and development. It is also a cofactor or required metal ion for many enzymes, such as superoxide dismutase, glutamine synthetase and arginase (Dukhande *et al.*, 2006). Synthesis, characterization and biological activity studies of the manganese complexes have become one of the most attractive research fields in modern bioinorganic chemistry.

In the title compound, the Mn(II) ion is coordinated with four oxygen atoms of the ciprofloxacin ligands in the equatorial positions while two oxygen atoms of the water occupy the axial positions resulting in a typical Jahn-Teller distorted octahedral geometry around the central metal atom. The Mn—O bond distances arising from the two carbonyl oxygen atoms O1 are longer,[2.153 (3) Å], than those arising from the carboxylate oxygen atoms O2, [2.122 (4) Å]. The axial average linkages between manganese and oxygen atoms of water are substantially longer [2.229 (4) Å] than the equatorial bond distances. The bond angles O1—Mn1—O1A, O2—Mn1—O2A and O1W—Mn1—O1W are 180° while the bond angles O2A—Mn1—O1W and O2—Mn1—O1W open up slightly from 89.17 (16)° to 90.83 (16)°, resulting in a slight distortion from the idealized octahedral geometry.

### Experimental

A mixture of 0.1 mmol ciprofloxacin hydrochloride, 0.1 mmol MnCl_2_.4H_2_O, 0.1 mmol 2,2'-bipyridine and 10 mL distilled water was sealed in a 25 mL Teflon-lined stainless vessel and heated at 433 K for 3 d, then cooled slowly to room temperature. The solution was filtered and after two weeks yellow single crystals were obtained.

### Refinement

The structure was solved by direct methods and successive Fourier difference synthesis. The H atoms bonded to C atoms were positioned geometrically and refined using a riding model [aromatic C—H = 0.93 Å, aliphatic C—H = 0.97 Å, methine C—H = 0.98 Å and N—H = 0.86 Å, *U*_iso_(H) = 1.2*U*_eq_(C),]. The H atoms bonded to O atoms were located in a difference Fourier maps and refined with O—H distance restraints of 0.85 (2) and *U*_iso_(H) = 1.5*U*_eq_(O).

### Crystal data

**Table:**

[Mn(C_17_H_17_FN_3_O_3_)_2_(H_2_O)_2_]·C_10_H_8_N_2_·4H_2_O	*Z* = 1
*M**_r_* = 979.89	*F*(000) = 513
Triclinic, *P*1	*D*_x_ = 1.436 Mg m^−^^3^
Hall symbol: -P 1	Mo *K*α radiation, λ = 0.71073 Å
*a* = 10.0355 (3) Å	Cell parameters from 2729 reflections
*b* = 11.1409 (3) Å	θ = 2.0–27.6°
*c* = 11.8461 (3) Å	µ = 0.37 mm^−^^1^
α = 66.905 (2)°	*T* = 296 K
β = 68.933 (2)°	Sheet, yellow
γ = 85.858 (2)°	0.42 × 0.17 × 0.05 mm
*V* = 1133.22 (6) Å^3^	

### Data collection

**Table:**

Bruker APEXII CCD area-detector diffractometer	5061 independent reflections
Radiation source: fine-focus sealed tube	3310 reflections with *I* > 2σ(*I*)
graphite	*R*_int_ = 0.042
φ and ω scans	θ_max_ = 27.6°, θ_min_ = 2.0°
Absorption correction: multi-scan (*SADABS*; Sheldrick, 1996)	*h* = −13→12
*T*_min_ = 0.926, *T*_max_ = 0.983	*k* = −14→14
14989 measured reflections	*l* = −15→15

### Refinement

**Table:**

Refinement on *F*^2^	Primary atom site location: structure-invariant direct methods
Least-squares matrix: full	Secondary atom site location: difference Fourier map
*R*[*F*^2^ > 2σ(*F*^2^)] = 0.084	Hydrogen site location: inferred from neighbouring sites
*wR*(*F*^2^) = 0.274	H atoms treated by a mixture of independent and constrained refinement
*S* = 1.08	*w* = 1/[σ^2^(*F*_o_^2^) + (0.1334*P*)^2^ + 2.3765*P*] where *P* = (*F*_o_^2^ + 2*F*_c_^2^)/3
5061 reflections	(Δ/σ)_max_ < 0.001
313 parameters	Δρ_max_ = 1.07 e Å^−^^3^
6 restraints	Δρ_min_ = −0.65 e Å^−^^3^

### Special details

**Table:**

Geometry. All e.s.d.'s (except the e.s.d. in the dihedral angle between two l.s. planes) are estimated using the full covariance matrix. The cell e.s.d.'s are taken into account individually in the estimation of e.s.d.'s in distances, angles and torsion angles; correlations between e.s.d.'s in cell parameters are only used when they are defined by crystal symmetry. An approximate (isotropic) treatment of cell e.s.d.'s is used for estimating e.s.d.'s involving l.s. planes.
Refinement. Refinement of *F*^2^ against ALL reflections. The weighted *R*-factor *wR* and goodness of fit *S* are based on *F*^2^, conventional *R*-factors *R* are based on *F*, with *F* set to zero for negative *F*^2^. The threshold expression of *F*^2^ > σ(*F*^2^) is used only for calculating *R*-factors(gt) *etc*. and is not relevant to the choice of reflections for refinement. *R*-factors based on *F*^2^ are statistically about twice as large as those based on *F*, and *R*- factors based on ALL data will be even larger.

### Fractional atomic coordinates and isotropic or equivalent isotropic displacement parameters (Å^2^)

**Table:**

	*x*	*y*	*z*	*U*_iso_*/*U*_eq_	
Mn1	0.0000	−0.5000	0.5000	0.0346 (3)	
F1	0.4111 (3)	−0.0038 (3)	−0.0828 (3)	0.0517 (9)	
N1	−0.1402 (4)	0.0159 (4)	0.2298 (4)	0.0313 (9)	
N2	0.3086 (4)	0.2389 (4)	−0.1295 (4)	0.0355 (9)	
N3	0.4635 (4)	0.4907 (4)	−0.2699 (4)	0.0406 (10)	
H3B	0.4905	0.5657	−0.2771	0.049*	
N4	0.1028 (5)	0.1554 (4)	0.3907 (5)	0.0447 (11)	
O1	0.0631 (4)	−0.3212 (3)	0.3262 (3)	0.0409 (9)	
O1W	−0.0267 (5)	−0.6032 (4)	0.3810 (4)	0.0494 (10)	
H1WA	0.004 (7)	−0.678 (3)	0.384 (6)	0.074*	
H1WB	−0.004 (8)	−0.564 (5)	0.297 (2)	0.074*	
O2	−0.2083 (4)	−0.4338 (3)	0.5255 (4)	0.0457 (9)	
O2W	0.3929 (3)	0.5957 (3)	−0.0476 (3)	0.0237 (6)	
H2WB	0.4451	0.5947	−0.0045	0.028*	
H2WA	0.445 (4)	0.601 (5)	−0.115 (2)	0.036*	
O3	−0.3858 (4)	−0.3070 (3)	0.5148 (4)	0.0490 (10)	
O3W	0.0870 (3)	−0.4804 (3)	0.0850 (3)	0.0279 (7)	
H3WA	0.0654	−0.4915	0.0265	0.034*	
H3WB	0.1756	−0.4736	0.0723	0.034*	
C1	−0.2563 (5)	−0.3264 (4)	0.4764 (5)	0.0339 (11)	
C2	−0.1559 (5)	−0.2151 (4)	0.3635 (5)	0.0313 (10)	
C3	−0.2132 (5)	−0.0963 (4)	0.3248 (5)	0.0328 (10)	
H3A	−0.3104	−0.0935	0.3682	0.039*	
C4	−0.2122 (5)	0.1368 (4)	0.2043 (5)	0.0344 (11)	
H4A	−0.2336	0.1699	0.1234	0.041*	
C5	−0.3182 (6)	0.1616 (5)	0.3180 (5)	0.0434 (13)	
H5A	−0.4015	0.2067	0.3057	0.052*	
H5B	−0.3349	0.0959	0.4058	0.052*	
C6	−0.1751 (6)	0.2373 (5)	0.2431 (5)	0.0423 (12)	
H6A	−0.1718	0.3285	0.1859	0.051*	
H6B	−0.1053	0.2178	0.2859	0.051*	
C7	0.0015 (5)	0.0152 (4)	0.1534 (4)	0.0294 (10)	
C8	0.0804 (5)	0.1295 (4)	0.0504 (5)	0.0313 (10)	
H8A	0.0372	0.2081	0.0333	0.038*	
C9	0.2217 (5)	0.1273 (4)	−0.0263 (4)	0.0307 (10)	
C10	0.2348 (5)	0.3576 (4)	−0.1651 (5)	0.0358 (11)	
H10A	0.2045	0.3884	−0.0943	0.043*	
H10B	0.1503	0.3394	−0.1792	0.043*	
C11	0.3359 (6)	0.4625 (5)	−0.2906 (5)	0.0388 (12)	
H11A	0.3639	0.4324	−0.3618	0.047*	
H11B	0.2872	0.5417	−0.3151	0.047*	
C12	0.5392 (6)	0.3696 (5)	−0.2344 (6)	0.0509 (15)	
H12A	0.6238	0.3877	−0.2204	0.061*	
H12B	0.5693	0.3391	−0.3054	0.061*	
C13	0.4387 (6)	0.2654 (5)	−0.1099 (6)	0.0473 (14)	
H13A	0.4868	0.1857	−0.0864	0.057*	
H13B	0.4124	0.2947	−0.0382	0.057*	
C14	0.2779 (5)	0.0040 (5)	−0.0004 (5)	0.0352 (11)	
C15	0.2065 (5)	−0.1080 (4)	0.1001 (5)	0.0333 (10)	
H15A	0.2499	−0.1866	0.1140	0.040*	
C16	0.0660 (5)	−0.1043 (4)	0.1832 (4)	0.0279 (9)	
C17	−0.0088 (5)	−0.2225 (4)	0.2956 (4)	0.0295 (10)	
C18	0.2345 (7)	0.2003 (6)	0.3002 (6)	0.0550 (15)	
H18A	0.2567	0.2904	0.2604	0.066*	
C19	0.3394 (7)	0.1219 (7)	0.2622 (6)	0.0551 (15)	
H19A	0.4289	0.1577	0.1983	0.066*	
C20	0.3059 (7)	−0.0116 (6)	0.3230 (6)	0.0551 (15)	
H20A	0.3739	−0.0680	0.3012	0.066*	
C21	0.1722 (6)	−0.0612 (6)	0.4157 (6)	0.0476 (13)	
H21A	0.1483	−0.1510	0.4553	0.057*	
C22	0.0728 (5)	0.0237 (5)	0.4498 (5)	0.0366 (11)	

### Atomic displacement parameters (Å^2^)

**Table:**

	*U*^11^	*U*^22^	*U*^33^	*U*^12^	*U*^13^	*U*^23^
Mn1	0.0330 (6)	0.0237 (5)	0.0326 (6)	−0.0009 (4)	−0.0045 (4)	−0.0028 (4)
F1	0.0347 (16)	0.0388 (17)	0.0486 (19)	0.0009 (13)	0.0122 (14)	−0.0083 (14)
N1	0.030 (2)	0.0248 (19)	0.028 (2)	0.0007 (15)	−0.0033 (16)	−0.0060 (16)
N2	0.032 (2)	0.027 (2)	0.032 (2)	−0.0046 (16)	−0.0048 (17)	−0.0012 (16)
N3	0.037 (2)	0.028 (2)	0.037 (2)	−0.0093 (17)	−0.0032 (19)	−0.0006 (18)
N4	0.043 (3)	0.041 (2)	0.041 (3)	−0.002 (2)	−0.006 (2)	−0.014 (2)
O1	0.0350 (19)	0.0264 (17)	0.038 (2)	0.0038 (14)	−0.0008 (15)	−0.0011 (14)
O1W	0.067 (3)	0.037 (2)	0.040 (2)	0.0014 (19)	−0.015 (2)	−0.0137 (17)
O2	0.0339 (19)	0.0291 (18)	0.046 (2)	−0.0016 (14)	−0.0031 (16)	0.0034 (15)
O2W	0.0189 (15)	0.0200 (14)	0.0395 (18)	0.0028 (11)	−0.0226 (13)	−0.0083 (13)
O3	0.0268 (19)	0.0349 (19)	0.053 (2)	−0.0019 (14)	0.0021 (16)	0.0004 (17)
O3W	0.0267 (16)	0.0398 (17)	0.0179 (15)	0.0073 (13)	−0.0140 (12)	−0.0074 (13)
C1	0.033 (3)	0.028 (2)	0.028 (2)	−0.0028 (19)	−0.002 (2)	−0.0058 (19)
C2	0.032 (2)	0.028 (2)	0.027 (2)	−0.0037 (18)	−0.0071 (19)	−0.0057 (18)
C3	0.025 (2)	0.029 (2)	0.030 (2)	−0.0051 (18)	−0.0009 (19)	−0.0032 (19)
C4	0.032 (3)	0.027 (2)	0.033 (3)	0.0026 (18)	−0.007 (2)	−0.0057 (19)
C5	0.042 (3)	0.037 (3)	0.042 (3)	0.010 (2)	−0.008 (2)	−0.014 (2)
C6	0.042 (3)	0.034 (3)	0.047 (3)	0.003 (2)	−0.012 (2)	−0.016 (2)
C7	0.025 (2)	0.028 (2)	0.026 (2)	−0.0016 (17)	−0.0043 (18)	−0.0055 (18)
C8	0.030 (2)	0.024 (2)	0.031 (2)	−0.0031 (18)	−0.004 (2)	−0.0072 (19)
C9	0.032 (2)	0.026 (2)	0.024 (2)	−0.0057 (18)	−0.0034 (19)	−0.0038 (18)
C10	0.033 (3)	0.027 (2)	0.035 (3)	−0.0016 (19)	−0.006 (2)	−0.005 (2)
C11	0.039 (3)	0.029 (2)	0.031 (3)	−0.006 (2)	−0.003 (2)	−0.002 (2)
C12	0.032 (3)	0.037 (3)	0.057 (4)	−0.005 (2)	−0.007 (3)	0.002 (3)
C13	0.034 (3)	0.033 (3)	0.051 (3)	−0.010 (2)	−0.013 (2)	0.007 (2)
C14	0.024 (2)	0.035 (3)	0.036 (3)	−0.0007 (19)	0.001 (2)	−0.013 (2)
C15	0.032 (3)	0.026 (2)	0.031 (3)	0.0012 (18)	−0.004 (2)	−0.0072 (19)
C16	0.027 (2)	0.023 (2)	0.026 (2)	−0.0028 (17)	−0.0045 (18)	−0.0044 (17)
C17	0.036 (3)	0.021 (2)	0.023 (2)	−0.0039 (18)	−0.0069 (19)	−0.0032 (17)
C18	0.051 (4)	0.046 (3)	0.050 (4)	−0.009 (3)	−0.006 (3)	−0.009 (3)
C19	0.040 (3)	0.069 (4)	0.044 (3)	−0.003 (3)	−0.001 (3)	−0.021 (3)
C20	0.045 (3)	0.060 (4)	0.045 (3)	0.003 (3)	0.000 (3)	−0.021 (3)
C21	0.049 (3)	0.044 (3)	0.043 (3)	0.003 (2)	−0.009 (3)	−0.017 (3)
C22	0.036 (3)	0.042 (3)	0.028 (3)	−0.004 (2)	−0.008 (2)	−0.013 (2)

### Geometric parameters (Å, °)

**Table:**

Mn1—O2^i^	2.122 (4)	C5—C6	1.495 (8)
Mn1—O2	2.122 (4)	C5—H5A	0.9700
Mn1—O1^i^	2.153 (3)	C5—H5B	0.9700
Mn1—O1	2.153 (3)	C6—H6A	0.9700
Mn1—O1W^i^	2.229 (4)	C6—H6B	0.9700
Mn1—O1W	2.229 (4)	C7—C8	1.403 (6)
F1—C14	1.363 (5)	C7—C16	1.410 (6)
N1—C3	1.342 (6)	C8—C9	1.386 (6)
N1—C7	1.386 (6)	C8—H8A	0.9300
N1—C4	1.458 (6)	C9—C14	1.408 (7)
N2—C9	1.413 (5)	C10—C11	1.526 (6)
N2—C10	1.456 (6)	C10—H10A	0.9700
N2—C13	1.476 (7)	C10—H10B	0.9700
N3—C11	1.463 (7)	C11—H11A	0.9700
N3—C12	1.487 (7)	C11—H11B	0.9700
N3—H3B	0.8600	C12—C13	1.516 (7)
N4—C18	1.343 (7)	C12—H12A	0.9700
N4—C22	1.356 (7)	C12—H12B	0.9700
O1—C17	1.273 (5)	C13—H13A	0.9700
O1W—H1WA	0.86 (2)	C13—H13B	0.9700
O1W—H1WB	0.86 (2)	C14—C15	1.356 (6)
O2—C1	1.255 (6)	C15—C16	1.409 (6)
O2W—H2WB	0.8500	C15—H15A	0.9300
O2W—H2WA	0.765 (18)	C16—C17	1.456 (6)
O3—C1	1.246 (6)	C18—C19	1.381 (9)
O3W—H3WA	0.8501	C18—H18A	0.9300
O3W—H3WB	0.8501	C19—C20	1.379 (9)
C1—C2	1.501 (6)	C19—H19A	0.9300
C2—C3	1.377 (6)	C20—C21	1.372 (8)
C2—C17	1.420 (7)	C20—H20A	0.9300
C3—H3A	0.9300	C21—C22	1.388 (7)
C4—C6	1.481 (7)	C21—H21A	0.9300
C4—C5	1.497 (7)	C22—C22^ii^	1.483 (10)
C4—H4A	0.9800		
			
O2^i^—Mn1—O2	180.00 (9)	N1—C7—C16	117.9 (4)
O2^i^—Mn1—O1^i^	83.55 (13)	C8—C7—C16	120.3 (4)
O2—Mn1—O1^i^	96.45 (13)	C9—C8—C7	121.2 (4)
O2^i^—Mn1—O1	96.45 (13)	C9—C8—H8A	119.4
O2—Mn1—O1	83.55 (13)	C7—C8—H8A	119.4
O1^i^—Mn1—O1	180.00 (19)	C8—C9—C14	116.5 (4)
O2^i^—Mn1—O1W^i^	90.83 (16)	C8—C9—N2	124.3 (4)
O2—Mn1—O1W^i^	89.17 (16)	C14—C9—N2	119.2 (4)
O1^i^—Mn1—O1W^i^	89.33 (14)	N2—C10—C11	109.3 (4)
O1—Mn1—O1W^i^	90.67 (14)	N2—C10—H10A	109.8
O2^i^—Mn1—O1W	89.17 (16)	C11—C10—H10A	109.8
O2—Mn1—O1W	90.83 (16)	N2—C10—H10B	109.8
O1^i^—Mn1—O1W	90.67 (14)	C11—C10—H10B	109.8
O1—Mn1—O1W	89.33 (14)	H10A—C10—H10B	108.3
O1W^i^—Mn1—O1W	180.0	N3—C11—C10	110.1 (4)
C3—N1—C7	119.8 (4)	N3—C11—H11A	109.6
C3—N1—C4	119.7 (4)	C10—C11—H11A	109.6
C7—N1—C4	120.4 (4)	N3—C11—H11B	109.6
C9—N2—C10	115.5 (4)	C10—C11—H11B	109.6
C9—N2—C13	113.4 (4)	H11A—C11—H11B	108.2
C10—N2—C13	110.5 (4)	N3—C12—C13	109.0 (4)
C11—N3—C12	109.6 (4)	N3—C12—H12A	109.9
C11—N3—H3B	125.2	C13—C12—H12A	109.9
C12—N3—H3B	125.2	N3—C12—H12B	109.9
C18—N4—C22	117.1 (5)	C13—C12—H12B	109.9
C17—O1—Mn1	128.5 (3)	H12A—C12—H12B	108.3
Mn1—O1W—H1WA	125 (4)	N2—C13—C12	110.3 (5)
Mn1—O1W—H1WB	121 (4)	N2—C13—H13A	109.6
H1WA—O1W—H1WB	98 (3)	C12—C13—H13A	109.6
C1—O2—Mn1	134.5 (3)	N2—C13—H13B	109.6
H2WB—O2W—H2WA	105.2	C12—C13—H13B	109.6
H3WA—O3W—H3WB	117.2	H13A—C13—H13B	108.1
O3—C1—O2	123.2 (4)	C15—C14—F1	117.9 (4)
O3—C1—C2	117.1 (4)	C15—C14—C9	124.0 (4)
O2—C1—C2	119.6 (4)	F1—C14—C9	118.1 (4)
C3—C2—C17	118.2 (4)	C14—C15—C16	119.3 (4)
C3—C2—C1	116.2 (4)	C14—C15—H15A	120.3
C17—C2—C1	125.5 (4)	C16—C15—H15A	120.3
N1—C3—C2	125.4 (4)	C15—C16—C7	118.3 (4)
N1—C3—H3A	117.3	C15—C16—C17	119.8 (4)
C2—C3—H3A	117.3	C7—C16—C17	121.9 (4)
N1—C4—C6	118.5 (4)	O1—C17—C2	125.9 (4)
N1—C4—C5	119.1 (4)	O1—C17—C16	117.9 (4)
C6—C4—C5	60.3 (4)	C2—C17—C16	116.1 (4)
N1—C4—H4A	115.9	N4—C18—C19	124.5 (6)
C6—C4—H4A	115.9	N4—C18—H18A	117.7
C5—C4—H4A	115.9	C19—C18—H18A	117.7
C6—C5—C4	59.3 (3)	C20—C19—C18	117.3 (6)
C6—C5—H5A	117.8	C20—C19—H19A	121.4
C4—C5—H5A	117.8	C18—C19—H19A	121.4
C6—C5—H5B	117.8	C21—C20—C19	119.9 (6)
C4—C5—H5B	117.8	C21—C20—H20A	120.1
H5A—C5—H5B	115.0	C19—C20—H20A	120.1
C4—C6—C5	60.4 (3)	C20—C21—C22	119.6 (5)
C4—C6—H6A	117.7	C20—C21—H21A	120.2
C5—C6—H6A	117.7	C22—C21—H21A	120.2
C4—C6—H6B	117.7	N4—C22—C21	121.6 (5)
C5—C6—H6B	117.7	N4—C22—C22^ii^	116.2 (6)
H6A—C6—H6B	114.9	C21—C22—C22^ii^	122.2 (6)
N1—C7—C8	121.7 (4)		
			
O2—Mn1—O1—C17	14.4 (4)	C13—N2—C10—C11	58.6 (6)
O1^i^—Mn1—O1—C17	−42 (43)	C12—N3—C11—C10	60.2 (5)
O1W^i^—Mn1—O1—C17	−74.7 (4)	N2—C10—C11—N3	−59.6 (5)
O1W—Mn1—O1—C17	105.3 (4)	C11—N3—C12—C13	−59.4 (6)
O1^i^—Mn1—O2—C1	165.3 (5)	C9—N2—C13—C12	169.4 (4)
O1—Mn1—O2—C1	−14.7 (5)	C10—N2—C13—C12	−59.1 (6)
O1W^i^—Mn1—O2—C1	76.1 (5)	N3—C12—C13—N2	58.6 (7)
O1W—Mn1—O2—C1	−103.9 (5)	C8—C9—C14—C15	−5.4 (8)
Mn1—O2—C1—O3	−173.9 (4)	N2—C9—C14—C15	176.6 (5)
Mn1—O2—C1—C2	7.6 (8)	C8—C9—C14—F1	173.1 (4)
O3—C1—C2—C3	8.7 (7)	N2—C9—C14—F1	−4.8 (7)
O2—C1—C2—C3	−172.7 (5)	F1—C14—C15—C16	−177.0 (4)
O3—C1—C2—C17	−171.9 (5)	C9—C14—C15—C16	1.6 (8)
O2—C1—C2—C17	6.6 (8)	C14—C15—C16—C7	3.9 (7)
C7—N1—C3—C2	6.1 (8)	C14—C15—C16—C17	−176.7 (5)
C4—N1—C3—C2	−176.1 (5)	N1—C7—C16—C15	175.4 (4)
C17—C2—C3—N1	−2.1 (8)	C8—C7—C16—C15	−5.5 (7)
C1—C2—C3—N1	177.3 (5)	N1—C7—C16—C17	−4.0 (7)
C3—N1—C4—C6	108.4 (5)	C8—C7—C16—C17	175.1 (4)
C7—N1—C4—C6	−73.9 (6)	Mn1—O1—C17—C2	−8.3 (7)
C3—N1—C4—C5	38.5 (7)	Mn1—O1—C17—C16	169.7 (3)
C7—N1—C4—C5	−143.8 (5)	C3—C2—C17—O1	173.5 (5)
N1—C4—C5—C6	108.1 (5)	C1—C2—C17—O1	−5.9 (8)
N1—C4—C6—C5	−109.1 (5)	C3—C2—C17—C16	−4.6 (6)
C3—N1—C7—C8	178.1 (5)	C1—C2—C17—C16	176.0 (4)
C4—N1—C7—C8	0.4 (7)	C15—C16—C17—O1	10.1 (7)
C3—N1—C7—C16	−2.8 (7)	C7—C16—C17—O1	−170.6 (4)
C4—N1—C7—C16	179.4 (4)	C15—C16—C17—C2	−171.7 (4)
N1—C7—C8—C9	−179.3 (4)	C7—C16—C17—C2	7.7 (7)
C16—C7—C8—C9	1.7 (7)	C22—N4—C18—C19	1.2 (9)
C7—C8—C9—C14	3.7 (7)	N4—C18—C19—C20	−0.7 (10)
C7—C8—C9—N2	−178.5 (4)	C18—C19—C20—C21	0.9 (10)
C10—N2—C9—C8	−8.4 (7)	C19—C20—C21—C22	−1.6 (10)
C13—N2—C9—C8	120.5 (5)	C18—N4—C22—C21	−1.8 (8)
C10—N2—C9—C14	169.4 (5)	C18—N4—C22—C22^ii^	179.4 (6)
C13—N2—C9—C14	−61.7 (6)	C20—C21—C22—N4	2.1 (9)
C9—N2—C10—C11	−171.1 (4)	C20—C21—C22—C22^ii^	−179.3 (6)

Symmetry codes: (i) −*x*, −*y*−1, −*z*+1; (ii) −*x*, −*y*, −*z*+1.

### Hydrogen-bond geometry (Å, °)

**Table:**

*D*—H···*A*	*D*—H	H···*A*	*D*···*A*	*D*—H···*A*
N3—H3B···O3^iii^	0.86	2.22	2.661 (5)	112
O1W—H1WA···N4^iv^	0.86 (2)	2.03 (2)	2.880 (6)	171 (7)
O3W—H3WB···O2W^iv^	0.85	2.13	2.910 (4)	153
O1W—H1WB···O3W	0.86 (2)	2.15 (2)	3.009 (5)	170 (6)
O2W—H2WA···N3	0.77 (2)	2.53 (3)	3.125 (5)	136 (4)

Symmetry codes: (iii) *x*+1, *y*+1, *z*−1; (iv) *x*, *y*−1, *z*.
